# Demographic and epidemiological characteristics of scorpion envenomation and daily forecasting of scorpion sting counts in Touggourt, Algeria

**DOI:** 10.4178/epih.e2020050

**Published:** 2020-07-06

**Authors:** Kaouthar Boubekeur, Mohamed L’Hadj, Schehrazad Selmane

**Affiliations:** 1 L’IFORCE, Faculty of Mathematics, University of Sciences and Technology Houari Boumediene, Bab Ezzouar, Algeria; 2 Health and Hospital Reform Services, Ministry of Health, Population and Hospital Reform, Algiers, Algeria

**Keywords:** Data analysis, Statistical models, Scorpion stings, Touggourt, Algeria

## Abstract

**OBJECTIVES:**

This study was conducted to provide better insights into the demographic and epidemiological characteristics of scorpion envenomation in an endemic area in Algeria and to identify the model that best predicted daily scorpion sting counts.

**METHODS:**

Daily sting data from January 1, 2013 to August 31, 2016 were extracted from questionnaires designed to elicit information on scorpion stings from the two emergency medical service providers in Touggourt, Algeria. Count regression models were applied to the daily sting data.

**RESULTS:**

A total of 4,712 scorpion sting cases were documented, of which 70% occurred in people aged between 10 years and 49 years. The male-to-female ratio was 1.3. The upper and lower limbs were the most common locations of scorpion stings (90.4% of cases). Most stings (92.8%) were mild. The percent of people stung inside dwellings was 68.8%. The hourly distribution of stings showed a peak between 10:00 a.m. and 11:00 a.m. The daily number of stings ranged from 0 to 24. The occurrence of stings was highest on Sundays. The incidence of scorpion stings increased sharply in the summer. The mean annual incidence rate was 542 cases per 100,000 inhabitants. The fitted count regression models showed that a negative binomial hurdle model was appropriate for forecasting daily stings in terms of temperature and relative humidity, and the fitted data agreed considerably with the actual data.

**CONCLUSIONS:**

This study showed that daily scorpion sting data provided meaningful insights; and the negative binomial Hurdle model was preferable for predicting daily scorpion sting counts.

## INTRODUCTION

Scorpion stings currently constitute a public health concern in many arid, semi-arid, or Saharan regions throughout the world within the stripe of 50° latitude, both south and north. Approximately 2 billion people are estimated to live in areas at risk for scorpion stings. Each year, an estimated 1.2 million people are victims of scorpion stings worldwide. Second only to snake-bites in terms of venomous animal-related human fatalities, scorpions are responsible for an estimated 3,000 deaths each year. However, these estimates are limited to the few countries that have a reliable system for scorpion sting epidemiological surveillance [1].

Owing to its climatic and geographic conditions and ecological characteristics, Algeria houses a diverse scorpion fauna [2]. Scorpion envenomations represent a major public health issue, with nearly three-quarters of the country’s population at risk for scorpion stings [3]. A national program for scorpion sting control has been operated since 1986 [4,5]. The yearly number of scorpion stings fluctuates around 50,000 cases, and scorpion stings therefore pose a heavy burden on the nation’s health care expenditures [2,6]. For the past ten years, the yearly number of deaths has been around 50, and most, if not all, fatalities were caused by *Androctonus australis* stings [7].

Several epidemiological surveys have been conducted in regions affected by scorpionism [8-10]. Most of the mathematical approaches intending to analyse the collected data are based on descriptive statistics [11,12]. The association of scorpion sting incidence with climate variables, using monthly data, in many affected regions, was previously performed using multiple linear regression [13-15]. However, in recent years, other statistical approaches using monthly data, such as times series analysis and count data, are taking over [16-19].

In this study, we scrutinised the demographic and epidemiological characteristics of scorpion stings in the Touggourt region and we presented a best fit model to forecast the daily scorpion sting counts in that region using climate variables. The approach used herein has been applied in several studies related to other health issues [20-25], but this is the first time it has been applied to daily scorpion stings.

## MATERIALS AND METHODS

### Study area

Touggourt lies in the Sahara, in the Righ valley region of northeastern Algeria, and is flanked by sand dunes and salt lakes to the north and south and small hills to the west. It is made up of 8 districts. The region is characterised by a harsh winter and a hot and dry summer.

### Scorpion sting data

Daily scorpion sting data from January 1, 2013 to August 31, 2016 were drawn from questionnaires designed to elicit information on scorpion stings from the 2 emergency service providers in the study region (a public hospital and a nearby public health centre in Touggourt). The questionnaires were anonymised. The extracted data included, inter alia, the following information: sex, age, date of sting, address, anatomical sting site, sting time, time of the first medical examination, evaluation on the first clinical examination, location (inside/outside the dwelling), and treatment administered. Data were analysed and graphs were generated to recognize trends and epidemiological and demographic features of scorpion stings using the software application Epi Info version 7.2.1.0 (http://www.cdc.gov/epiinfo/).

### Weather data

Daily data on the average temperature (T) and relative humidity (RH), accumulated rainfall, wind speed, and evaporation value from January 1, 2013 to August 31, 2016, was supplied by the Touggourt meteorological station.

### Modelling method

Count data occur in many fields, including public health and epidemiology. To handle count data, various models have been developed, including Poisson, zero-inflated Poisson (ZIP), Poisson hurdle (PH), negative binomial (NB), zero-inflated negative binomial (ZINB), and negative binomial hurdle (NBH) models. The Poisson model is the most widely used model for count events occurring within a specific period. A key feature of the Poisson model is equality between the mean and the variance; however, this equality does not always occur when dealing with real-world data. Indeed, real-world data are often characterised by overdispersion, which occurs when the variance exceeds the mean. When overdispersion is present, Poisson regression is usually given up in favour of NB regression, which adjusts for overdispersion. However, the NB model can only take into account overdispersion due to unobserved heterogeneity in the data, not overdispersion arising from an excess of zeroes. To handle overdispersion that results only from an excess of zeroes, zero-inflated Poisson and hurdle Poisson models are used. To account for overdispersion from an excess of zeroes and unobserved heterogeneity, zero-inflated NB or hurdle NB models are preferred given their flexibility [26-29]. The Supplementary Material 1 presents a detailed description of each involved model, as well as the various statistical tests used to assess overdispersion, to compare the models under consideration, and to select the best model. We briefly review only the model that was found to be the most appropriate to forecast the daily stings in terms of climate variables.

The hurdle model is a 2-component model; a hurdle component used to model large zero counts, and a truncated count component used to model only positive counts. The hurdle model can be expressed as follows:

$$P(Y_{i}\;=\;y_{i}|x_{i},\;z_{i},\;\beta ,\;\gamma )\;=\left\{\begin{array}{l} f_{zero}\;(0;\;z_{i},\;\gamma )\\ \frac{1\;-\;f_{zero}\;(0;\;z_{i},\;\gamma )}{1\;-\;f_{count}\;(0;\;x_{i},\;\beta )}f_{count}\;(y_{i};\;x_{i},\;\beta )\;=\;\Phi \;f_{count}\;(y_{i};\;x_{i},\;\beta ) \end{array}\right.\;\begin{array}{c} if\;y_{i}\;=\;0\\ if\;y_{i}\;>\;0 \end{array}$$

where *y* is the dependent variable, *x* is a vector of covariates for positive counts and *z* is a vector of covariates in the zero part. The model parameters *β, γ* are related to *x* and z, respectively, and are estimated by maximum likelihood. *f_zero_* is a probability density function at least on {0, 1} or {0, 1, 2, …}, and *f_count_* is a probability density function on {0, 1, 2, …}. The *f_zero_* part (where *y_i_*=0) is modelled using a binary logit (logistic regression) model, where all positive counts are given a value of 1. The probability of *y_i_*=0 is given by

$$f_{zero}\;(0;\;z_{i},\;\gamma )\;=\;\frac{1}{1\;+\;e^{z_{i}\gamma }}$$

The probability of a non-zero count is therefore given by 1 - *f_zero_* (0; *z_i_*, *γ*). Regarding *f_count_* (*y_i_*; *x_i_*, *β*), it is modelled with a left-truncated (*y_i_*>0) Poisson model or negative binomial model in case of overdispersion with log link. The corresponding mean regression relationship is given by

$$\log(\mu_{i})\;=\;x_{i}^{T}\beta \;+\;\log(1-f_{zero}\;(0;\;z_{i},\;\gamma ))\;-\;\log(1\;-\;f_{count}\;(0;\;x_{i},\;\beta )).$$

Some climate variables have been shown to affect the count of monthly scorpion stings [3,13,14], so we considered incorporating the available climate variables at the Touggourt meteorological station into the models. In order to take into account other factors that may contribute to scorpion sting accidents, a trend variable (Tr) was incorporated into the models. The scatter plot, along with the Pearson product-moment correlation coefficient between the scorpion sting variable, S, and each one of the climate variables was used as a guide to select the appropriate climate covariates.

The 6 regression models outlined in the Supplementary Material 1 were fitted to the data. The Akaike information criterion (AIC) and Bayesian information criterion (BIC) were estimated to assess the goodness-of-fit of the 6 models. The model with the minimum AIC and BIC values is preferred [26]. To compare and test the goodness-of-fit between model pairs, likelihood ratio tests and Vuong statistics were performed. Then, to evaluate the 6 models, the mean absolute error (MAE) and root mean squared error (RMSE) were estimated to measure the closeness of the observed values to the predicted values [28].

The RStudio version 1.1.383 (https://www.npackd.org/p/rstudio/1.1.383) was used to perform all analyses and modelling [30-32]. The R code for mapping the incidence and the R code for fitting and forecasting the daily scorpion sting data are provided in Supplementary Material 1.

### Ethics statement

This study was approved by the Committee of National Scorpionism Control Program. The proposal was sent to the Ethics Committee on January 2, 2017. The proposal was approved on February 6, 2017.

## RESULTS

### Spatial distribution of scorpion sting incidence in different districts of Touggourt

**Figure 1.**
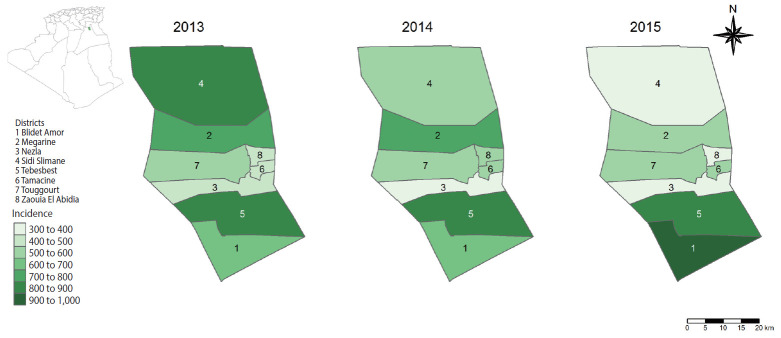
Spatial distribution map of scorpion sting incidence per 100,000 inhabitants in Touggourt, for the years 2013, 2014, and 2015.

Figure 1 depicts the spatial distribution per district of scorpion sting incidence per 100,000 population for the years 2013, 2014 and 2015. An increase of scorpion sting incidence over time was observed in the Blidet Amor district, a decrease was observed in the Sidi Slimane district, and slight fluctuations prevailed in the remaining 6 districts. The annual incidence rate was 561 cases per 100,000 inhabitants in 2013, 541 cases per 100,000 inhabitants in 2014, and 523 cases per 100,000 inhabitants in 2015. At the district level, the incidence ranged between 341 and 977 per 100,000 inhabitants, dramatically exceeding the mean national incidence rate, which has been estimated as 125 per 100,000 inhabitants [6]. The correlation between the number of scorpion stings per district and the size of the population was high; the Pearson product-moment correlation coefficient was 0.91 in 2013, 0.81 in 2014, and 0.75 in 2015.

### Epidemiological features of scorpion envenomations

From January 1, 2013 to August 31, 2016, a total of 4,712 scorpion sting victims, including 7 scorpion-related human fatalities, were documented at the 2 emergency service providers in Touggourt. Table 1 gives a breakdown of registered cases by sex, by age group, by anatomical sting site, by the evaluation on the first clinical examination, by sting time, and by location. The victims were predominantly male (56.4%). Children under the age of 15 accounted for about one-fifth of the stung people (19.9%), and the population in the active age (18-64 years) was (as expected) the most frequently stung (68.7%). Of the sting victims, 57.2% were male and 42.8% female, and the elderly (aged 65 or higher) represented 7.5% of cases. The mean ± standard deviation age was 32.02± 19.05 years (95% confidence interval [CI], 31.47 to 32.57). The limbs were the body part most exposed to scorpion stings, accounting for 91.3% of cases; 52.8% of stings (of which 40.5% were in females and 59.5% in males) affected the lower limbs and 38.5% (of which 47.7% were in females and 52.3% in males) affected the upper limbs. The percentage of people sting inside dwellings was 70.7% (of which 51.4% were in females and 48.6% in males), with the highest recorded frequency between 10 a.m. and 11 a.m. In the 29.3% of cases where a person was stung outside of his or her dwelling, there was a strong male predominance (75.8% of these cases were in males and 24.2% in females) with the highest recorded frequency between 10 a.m. and 11 a.m. Medical care was performed in a timely manner; 92.7% of victims received medical assistance within 2 hours following the sting accident, and 54.0% did so in less than 1 hour.

On the first medical examination, 4,371 (92.8%) cases were classified as mild, with a male-to-female ratio of 1.3, 187 (4.0%) cases as moderate, with a male-to-female ratio of 1.4, and 7 cases as severe, of which 5 occurred in females. For 147 cases (3.1%), that information was not provided. Antivenom was used in 4,455 (94.5%) patients. Seven scorpion-related human fatalities occurred (3 males and 4 females). At the first medical exam, 5 of those cases were classified as severe. The victims were aged, respectively 4, 15, 26, 30, 39, 80, and 88. All of them were stung inside their dwelling and on the limbs (the lower limbs in 5 cases).

**Figure 2.**
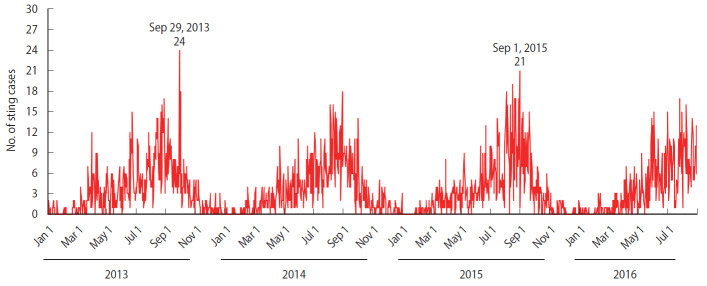
Frequencies of daily scorpion stings in Touggourt from January 1, 2013 to August 31, 2016.

**Figure 3.**
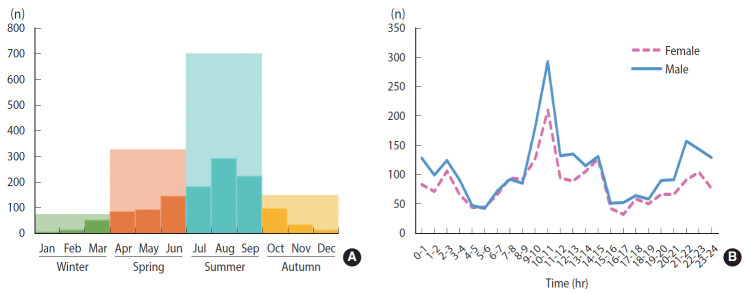
(A) Seasonal average and monthly average of recorded scorpion sting cases in Touggourt in the period 2013-2015 and (B) the hourly breakdown of scorpion sting cases.

Stings occur year-round, as depicted in Figure 2. Over the 1,339-day coverage of the study period, no stings were recorded by the emergency services on 320 days. For the years 2013-2015, more than half of the sting cases were recorded during the summer, followed by the spring, then the autumn and the winter (Figure 3A). August had the highest monthly frequency of events, followed by September and then July (Figure 3A). The hourly distribution of stings, displayed in Figure 3B, shows a peak between 9 a.m. and 11 a.m. (17.6% of cases) and off-peaks at 5 a.m. and at 4 p.m. The most daily stings occurred on September 29, 2013, with 24 sting cases (including 20 stings that occurred inside dwellings), followed by 21 sting cases (including 19 stings that occurred inside dwellings) on September 1, 2015 and 19 sting cases (including 16 stings that occurred inside dwellings) on August 18, 2015 (Figure 2). These daily peaks all involved mild cases.

### Statistical modelling output

The dataset included 1,339 observations and was divided into a training set, which included 1,095 (82.0%) observations from January 1, 2013 to December 31, 2015 to estimate the parameters of the best model, and a test set that included 244 (18.0%) observations from January 1, 2016 to August 31, 2016 to evaluate the model.

An examination of the relationship between monthly scorpion sting data and the climate variables showed a strong correlation with monthly average temperature (r = 0.91) with an apparent quadratic relationship (S= -0.0004 T^2^+0.19 T+9.73; R^2^= 0.95) and high correlation with relative humidity (r= -0.70) with a quadratic relationship (S= 0.0007 RH^2^ -0.29 RH+61.74; R^2^= 0.72); which is consistent with results obtained in other geographical regions affected by scorpionism [16,17]. At the daily level, the relationship was not readily apparent, although a high correlation was found for T (r= 0.69) and the correlation with RH was significant (r= -0.45); however, these climate variables were statistically significant in the modelling process.

We started by fitting the Poisson model with S, the daily scorpion sting count, as a dependent variable and all climate variables and trends as covariates. Drawing on the likelihood ratio test, only average T, RH, and Tr were withheld in the final fitted standard Poisson regression model.

As the variance (14.78) was 4 times higher than the mean (3.52), indicating overdispersion, and close to quarter of the observations were zeroes (23.9%), regression models accounting for both overdispersion and an excess of zeroes were considered. Hence, the ZIP, PH, NB, ZINB, and NBH models were fitted to the daily scorpion sting count using the same covariates as for Poisson model. Table 2 displays the statistical tests used to compare and test the goodness-of-fit of the 6 models. NB-type models (NB, ZINB, and NBH) had smaller AIC and BIC values than the Poisson-type models (Poisson, ZIP, and PH). Likewise, the likelihood ratio *χ*^2^ was highly significant for all models and the NB-type models had smaller values than the Poisson-type models. Moreover, the NBH model fitted the daily scorpion sting data better than all the other models. NB-type models are preferred to Poisson-type models to handle the overdispersion of the daily scorpion sting counts, and the statistical tests indicate that overdispersion was due to both heterogeneity and excess zeroes. Moreover, the Vuong test indicated that both the zero-inflated models and hurdle models were better and more effective at handling the excessive zero counts than the Poisson and NB models. Finally, the test set was used to evaluate the models. The RMSE and MAE were estimated. The MAE was smaller for predicting the frequency of stings from January to March using the Poisson model and from April to August using the NBH model. These findings therefore suggest that the NBH model is preferable for modelling the daily scorpion sting count using the considered criteria.

For brevity, only the NBH model estimates are presented. The estimated parameters, standard errors, and associated p-values are displayed in Table 3. The NBH model consists of two parts.

The zero part contains information about variables that the nested logistic regression model used to estimate the probability (ϕ) of observing a zero count:

logit(S = 0) = -3.1503 + 0.1556 * T + 0.0022 * RH + 2.4777 * Tr

Notice that the logistic regression model did not find RH to be a useful variable for estimating ϕ. Indeed, the regression coefficient was found to be not statistically significant at the 95% CI, as indicated by the p-value (0.81) which is greater than 0.05. The 2 variables that the logistic regression model determined to be useful for estimating the probability of observing a zero count were T and Tr.

The count part contains information about the variables that the NBH model used to estimate scorpion sting count on the condition that S>0.

log(S) = -2.4052 + 0.1129 * T + 0.0148 * RH + 0.1242Tr

The coefficients for T and RH were statistically significant, as evidenced by their respective p-values. The coefficients for T and RH are positive, meaning that as T and RH went up, the number scorpion sting increased.

**Figure 4.**
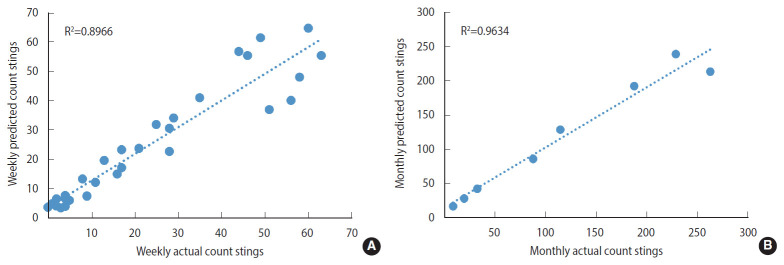
Scatter plots of predicted versus actual of scorpion sting cases from January to August 2016, (A) weekly data, (B) monthly data.

By aggregating the estimated daily data for the validation period into weekly data and monthly data, the predicted data were strongly correlated with the actual data (Pearson product-moment correlation coefficient: r= 0.94 for weekly data and r= 0.98 for monthly data) as shown in Figure 4, confirming the appropriateness and effectiveness of the NBH model to predict scorpion stings in terms of climate variables with very high accuracy.

## DISCUSSION

The purpose of this paper was, first, to provide better insights into the demographic and epidemiological characteristics of scorpion stings in an endemic region in Algeria, and second, to model the daily scorpion sting count as a function of climate factors using 6 count data models and to compare the performance of these models.

The epidemiological analysis showed a male predominance, following a similar pattern to that at the national level [6]. Similar findings have been documented in Morocco and Iran [9,33]. However, this is not a strict rule, as a study carried out in the Rio Grande do Norte state in Brazil showed a female predominance [34]. The findings must be interpreted cautiously, as they reflect a stratification by sex of the number of scorpion stings, and not a stratification by sex of the incidence rate. The most prone body parts to stings were found to be the legs and arms. Several studies carried out in other affected provinces of the country and in other affected countries have presented consistent findings [8,9,16,17,33]. The most likely reason that moving body parts are at a greater risk of scorpion stings is the fact that many victims do not protect themselves to avoid stings during their activities; they bear a significant share of responsibility for these accidents, either through ignorance or negligence. With respect to location, a significant variation according to the sex of the patient was observed, and almost three-quarters of sting accidents occurred inside dwellings. The percentage of people stung inside dwellings differs from province to province; in Biskra Province, situated in the central-eastern area, 46% of sting cases occurred inside dwellings, whereas in El Bayadh Province, in western Algeria, 65% of sting cases arose inside dwellings [16,17]. The high frequency of accidents inside the dwelling may reflect significant exposure to scorpion accidents in home environment while carrying out domestic activities. Moreover, due to the high temperatures in the Saharan region, people spend the majority of their waking hours inside their dwellings. Ninety percent of sting accidents occurred between April to October, corresponding to the hottest months (July and August), which recorded the highest frequency of accidents, followed by the date harvest season (September-October) and the planting season (April-May) where scorpion sting accidents are common on farms. The high incidence in summer has been observed in various regions affected by scorpionism [9,33]. Several studies in different geographical regions have documented varied age-class distributions of stings, thereby corroborating the geographical variation within epidemiological indicators that was noted in the global appraisal on the epidemiology of scorpionism by Chippaux & Goyffon [1]. Individuals aged 20-29 years old made up the largest number of cases, accounting for 24% of all cases; this was also reported in the Rio Grande do Norte State in Brazil [34]. Sting cases were predominantly mild and progressed towards recovery. Similar results were reported in other regions [8-10]. The time interval until first medical care was less than 3 hours after the victim for most victims, revealing that the population has become aware of the need to seek care right after the sting and that public awareness campaigns to avoid the sting itself have failed. Due to the seasonal pattern of scorpion stings, launching regular public awareness-raising campaigns in high-risk regions during the peak months might significantly reduce the number of sting incidents.

In prior studies, modelling of scorpion stings was performed with monthly data using statistical approaches such multiple linear regression, time series analysis, and count data [13-17]. No modelling of daily observations has been conducted to date. Aside from delivering relevant insights, modelling on the basis of daily data could produce highly accurate predictions, provided the availability of accurate forecasts of the climate variables.

In this study, for the first time, scorpion sting data were analysed without recourse to monthly aggregation as has been done in the literature to date. The fitted count regression models using the daily scorpion sting count as the dependent variable, and T, RH, and trends therein as independent variables showed that the NBH model yielded adequate fit. T and RH affected sting accidents, and T had the strongest effect. Additionally, daily predictions by means of the NBH model provided highly accurate monthly forecasts. These predictions could assist public health decision-makers in initiating appropriate, rapid, and effective measures and in containing any unusual situations.

A major contribution of this study is the improvement in the forecasting accuracy of scorpion sting cases in terms of weather conditions. However, in view of the impact of scorpion envenomation in both financial and human terms, more research is needed to define alternative policies to avoid sting accidents rather than accepting them as inevitable. This novel study on the daily scorpion sting count, in terms of the statistical approach used and the results that were obtained, is a contribution towards literature and the modelling of scorpion stings that encourages further investigations and studies of scorpionism.

## Figures and Tables

**Table 1.** Demographic and epidemiological characteristics of patients stung by scorpions from January 1, 2013 to August 31, 2016

Characteristics	Female	Male	Male/Female	Total
Age (yr)				
0-9	200	218	1.1	418
10-19	370	511	1.4	881
20-29	437	677	1.5	1,114
30-39	355	431	1.2	786
40-49	227	272	1.2	499
50-59	198	197	1.0	395
60-69	120	158	1.3	278
70-79	57	98	1.7	155
>80	27	40	1.5	67
Unknown	65	54	0.8	119
Anatomical sting site				
Lower limbs	998	1,467	1.5	2,465
Upper limbs	855	939	1.1	1,794
Head/neck	120	113	0.9	233
Trunk	65	108	1.7	173
Unknown	18	29	1.6	47
Grade on first clinical examination				
Mild	1,910	2,461	1.3	4,371
Moderate	77	110	1.4	187
Severe	5	2	0.4	7
Unknown	64	83	1.3	147
Sting time				
0:00-5:59	414	531	1.3	945
6:00-11:59	683	854	1.3	1,537
12:00-17:59	453	548	1.2	1,001
18:00-23:59	453	668	1.5	1,121
Unknown	53	55	1.0	108
Location				
Inside dwellings	1,667	1,575	0.9	3,242
Outside dwellings	325	1,020	3.1	1,345
Unknown	64	61	1.0	125

**Table 2.** AIC, BIC, LR chi-square, LRT, and Vuong test results of the models

Variables	Poisson	NB	ZIP	ZINB	PH	NBH
AIC	4,472.9	4,240.1	4,393.2	4,216.8	4,392.1	4,208.9
BIC	4,492.9	4,265.1	4,433.2	4,261.8	4,432.1	4,253.9
LR χ^2^	4,464.9	4,230.1	4,377.2	4,198.8	4,376.1	4,190.9
χ^2^ p-value	<0.001	<0.001	<0.001	<0.001	<0.001	<0.001
LRT	NB-P	ZINB-ZIP	NBH-PH	P-ZIP	NB-ZINB	
	234.83	178.44	185.23	87.68	31.28	
p-value	<0.001	<0.001	<0.001	<0.001	<0.001	
Vuong	ZIP-P	ZINB -NB	PH-P	NBH-NB	PH-ZIP	NBH-ZINB
	4.07	2.79	4.07	3.05	0.28	1.2
p-value	<0.001	0.003	<0.001	0.001	0.388	0.114

AIC, Akaike information criterion; BIC, Bayesian information criterion; LR, likelihood ratio; LRT, likelihood ratio test; NB, negative binomial; ZIP, zero-inflated Poisson; ZINB, zero-inflated negative binomial; P, Poisson; PH, Poisson hurdle; NBH, negative binomial hurdle.

**Table 3.** Estimated parameters, SE, and associated p-values for the NBH model

Factor	Zero part			Count part		
	Estimate	SE	p-value	Estimate	SE	p-value
Intercept	-3.150	0.725	<0.001	-2.405	0.253	<0.001
T	0.156	0.027	<0.001	0.113	0.007	<0.001
RH	0.002	0.009	0.807	0.015	0.003	<0.001
Tr	2.478	0.640	<0.001	0.124	0.220	0.572

SE, standard error; NBH, negative binomial hurdle; T, time; RH, relative humidity; Tr, trend.

## References

[1] Chippaux JP, Goyffon M (2008). Epidemiology of scorpionism: a global appraisal. Acta Trop.

[2] Vachon M (1952). Etudes sur les scorpions. http://www.ntnu.no/ub/scorpion-files/vachon.php.

[3] Selmane S (2015). Scorpion envenomations and climate conditions: the case of Naama Province in Algeria. Int J Math Models Methods Appl Sci.

[4] Ministry of Health, Population and Hospital Refor (2009). National committee for the fight against the scorpion envenomment. Support for scorpion envenomation.

[5] Ministry of Health, Population and Hospital Reform, Prevention Department Instruction no 04 / MSPRH / DP of March 4, 2012 on the new plan of notification and management of scorpionic envenomation cases. http://www.sante.dz/inst_env_scorp.pdf.

[6] Laïd Y, Boutekdjiret L, Oudjehane R, Bachiri K (2000-2014). Scorpion envenomation: the annual report on the epidemiological situation in Algeria. http://www.insp.dz/index.php/envenimation-scorpionique.

[7] National Institute of Public Health (2011). Epidemiological characteristics of deaths from scorpionic envenomation in Algeria. http://insp.dz/index.php/Non-categorise/envenimation-scorpionique.html.

[8] Selmane S, Benferhat L, L’Hadj M, Zhu H (2017). Scorpionism in Sidi Okba, Algeria: a cross-sectional study of 2016 stung patients between 2014 and 2015. Trop Biomed.

[9] Elyamani R, Soulaymani A, Serheir Z (2019). Epidemiology of scorpion envenoming in the prefecture of Figuig, Morocco. Int J Med Toxicol Forensic Med.

[10] Ozkan O, Uzun R, Adiguzel S, Cesaretli Y, Ertek M (2008). Evaluation of scorpion sting incidence in Turkey. J Venom Anim Toxins Incl Trop Dis.

[11] Nejati J, Saghafipour A, Rafinejad J, Mozaffari E, Keyhani A, Abolhasani A (2018). Scorpion composition and scorpionism in a high-risk area, the southwest of Iran. Electron Physician.

[12] El Hidan MA, Touloun O, El Oufir R, Boumezzough A (2016). Epidemiological and spatial analysis of scorpion stings in two regions of Morocco: Marrakesh-Tensift-Al Haouz and Souss-Massa-Draa. J Coast Life Med.

[13] Chowell G, Hyman JM, Díaz-Dueñas P, Hengartner NW (2005). Predicting scorpion sting incidence in an endemic region using climatological variables. Int J Environ Health Res.

[14] Selmane S, L’hadj M (2014). Regression analysis on scorpion envenomation and climate variables in M’Sila province, Algeria from 2001 to 2010. Int J Math Trends Technol.

[15] Selmane S, El Hadj H, Benferhat L (2014). The impact of climate variables on the incidence of scorpion stings in humans in M’Sila’s Province in Algeria.

[16] Selmane S, L’Hadj M (2016). Forecasting and prediction of scorpion sting cases in Biskra province, Algeria, using a seasonal autoregressive integrated moving average model. Epidemiol Health.

[17] Selmane S, Benferhat L, L’Hadj M, Zhu H (2016). Modelling the scorpion stings using surveillance data in El Bayadh Province, Algeria. Asian Pac J Trop Dis.

[18] Ebrahimi V, Hamdami E, Moemenbellah-Fard MD, Ezzatzadegan Jahromi S (2017). Predictive determinants of scorpion stings in a tropical zone of south Iran: use of mixed seasonal autoregressive moving average model. J Venom Anim Toxins Incl Trop Dis.

[19] Kassiri H, Shemshad K, Kassiri A, Shemshad M, Valipor AA, Teimori A (2013). Epidemiological and climatological factors influencing on scorpion envenoming in Baghmalek County, Iran. Acad J Entomol.

[20] Chipeta MG, Ngwira BM, Simoonga C, Kazembe LN (2014). Zero adjusted models with applications to analysing helminths count data. BMC Res Notes.

[21] Cheung YB (2002). Zero-inflated models for regression analysis of count data: a study of growth and development. Stat Med.

[22] Lee JH, Han G, Fulp WJ, Giuliano AR (2012). Analysis of overdispersed count data: application to the Human Papillomavirus Infection in Men (HIM) Study. Epidemiol Infect.

[23] Rose CE, Martin SW, Wannemuehler KA, Plikaytis BD (2006). On the use of zero-inflated and hurdle models for modeling vaccine adverse event count data. J Biopharm Stat.

[24] Tüzen MF, Erbaş S (2018). A comparison of count data models with an application to daily cigarette consumption of young persons. Commun Stat Theory Methods.

[25] Sarvi F, Moghimbeigi A, Mahjub H, Nasehi M, Khodadost M (2019). Factors associated with mortality from tuberculosis in Iran: an application of a generalized estimating equation-based zero-inflated negative binomial model to national registry data. Epidemiol Health.

[26] Cameron AC, Trivedi PK (2013). Regression analysis of count data.

[27] Hilbe JM (2011). Negative binomial regression.

[28] Hilbe JM (2014). Modeling count data.

[29] McCullagh P, Nelder JA (1989). Generalized linear models.

[30] Beaujean AA, Grant MB (2016). Tutorial on using regression models with count outcomes using R. Pract Assess Res Eval.

[31] Friendly M, Meyer D, Zeileis A (2015). Discrete data analysis with R: visualization and modeling techniques for categorical and count data.

[32] Zeileis A Kleiber C, Jackman S (2008). Regression models for count data in R. J Stat Softw.

[33] Ghorbani E, Mohammadi Bavani M, Jafarzadeh S, Saghafipour A, Jesri N, Moradiasl E (2018). Spatial distribution of scorpionism in Ardabil Province, North West of Iran. Int J Pediatr.

[34] Araújo KA, Tavares AV, Marques MR, Vieira AA, Leite RS (2017). Epidemiological study of scorpion stings in the Rio Grande do Norte State, Northeastern Brazil. Rev Inst Med Trop Sao Paulo.

