# Supplementary material for: Demographic and epidemiological characteristics of scorpion envenomation and daily forecasting of scorpion sting counts in Touggourt, Algeria
Source: Epidemiol Health. 2020 Jul 6;42:e2020050. doi: 10.4178/epih.e2020050 (PMC7871152; doi:10.4178/epih.e2020050)
Supplement: Supplementary Material 1. [file epih-42-e2020050-suppl.pdf]

Poisson

Poisson regression is commonly used in public health to model the number of events (y)or rate (r). It's often assumed that the number of events follows a Poisson distribution with a conditional mean (μ) depending upon a set of regressors (x) and corresponding parameters (β) for a participant's linear predictor. Using a log link we can express the expected number of events for participant i at dose j as
$\mu_{ij} = E(y_{ij}|x_{ij}) = exp(\beta' x_{ij})$. The Poisson probability distribution of y_{ij} given x_{ij} can be expressed as

$$\Pr(Y_{ij} = y_{ij}) = \frac{\mu_{ij}^{y_{ij}}}{y_{ij}!} exp(-\mu_{ij}) \qquad (1)$$

where y is a non-negative integer. The contribution of the ith participant at dose j to the log-likelihood for the Poisson model can be expressed as

$$LL(\beta) = y_{ij}(\beta x_{ij}) - exp(\beta x_{ij}) - ln(y_{ij}!)$$

The log-likelihood of all other considered models can be expressed using their respective distribution. A property of Poisson regression, one often violated, is that the variance equals the mean, i.e., $Var(y) = \sigma^2 = \mu$. If over-dispersion is an issue (i.e., variance exceeds the mean) the estimated parameters based on Poisson regression will be inefficient (Cameron and Trivedi, 1998). Generally, over-disperion is caused by unobserved heterogeneity, temporal dependency, and/or excess zeroes
in the data.

Negative Binomial

We can relax the variance assumption of Poisson regression and allow for an over-dispersion parameter by using the Negative Binomial model. Over-dispersion may be accounted for when using the NB model because of the addition of an error term, e, to the conditional mean of the Poisson regression model (Sheu et al., 2004), i.e., $\mu = exp(\beta' x_{ij} + e_{ij})$. We normally assume that $exp(e_{ij})$ has a gamma distribution
with mean 1 and variance a so that the conditional mean of y_{ij} is still μ_{ij} but the conditional variance of y_{ij} becomes $\mu_{ij}(1 + a\mu_{ij})$. As a approaches zero, y becomes a Poisson distribution and as a becomes larger the distribution becomes more dispersed. The NB probability distribution for participant i at dose j is given by:

$$P(Y_{ij} y_{ij}) = \frac{\Gamma(y_{ij} + 1/a)}{\Gamma(y_{ij} + 1)\Gamma(1/a)} \frac{(a\mu_{ij})^{y_{ij}}}{(1 + a\mu_{ij})^{y_{ij}+1/a}} \qquad (2)$$

Where μ_{ij} , a, and $\Gamma(\bullet)$ refer to the mean of the count distribution, the NB dispersion parameter, and the gamma function. The NB model is generally adequate for addressing over-dispersion due to unobserved heterogeneity and/or temporal dependency, but may be inadequate for over-dispersion resulting from excess zeroes.

In recent years, zero-inflated and hurdle models have gained popularity for modeling count data with excess zeroes. According to Cameron and Trivedi (1998), zero-inflated and hurdle models can be viewed as finite mixture models with a degenerate distribution whose mass is concentrated at zero. Excess zeroes arise when the event of interest is not experienced by many of the subjects.

Zero-Inflated Poisson

The zero-inflated Poisson distribution for participant i at dose j can be defined as

$$\Pr(Y_{ij} = y_{ij}) = \begin{cases} p_{ij} + (1 - p_{ij}) \exp(-\mu_{ij}), & y_{ij} = 0 \\ (1 - p_{ij}) \dfrac{\mu_{ij}{}^{y_{ij}}}{y_{ij}!} \exp(-\mu_{ij}), & y_{ij} = 0 \end{cases} \quad (3)$$

The probability of being an excess zero (p_{ij} in Eq. (3) is often modeled using logistic regression. Here, for all zero-inflated and hurdle models, we use the logistic model to estimate p_{ij}. Hence, p_{ij} is estimated using

$$p_{ij} = \frac{1}{1 + \exp(-\eta_{ij})} \quad (4)$$

Where η_{ij} is related to a set of explanatory variables $(\boldsymbol{x})$. Zero-inflated models put more weight on the probability of observing a zero by using a mixing distribution. Hence, for ZIP model (3) the probability of observing a zero is given by the sum of observing an excess zero plus the probability of observing a zero in the Poisson model. As illustrated, the ZIP model allows for two separate processes. Conceptually, the first step models the structural zeroes (e.g., logistic regression) and the second step models the Poisson distribution conditional on the excess zeroes, i.e., Poisson regression models the sampling zeroes and counts. The mean and variance of the ZIP model are given by

$$E(y) = (1 - p)\mu$$
$$Var(y) = \mu(1 - p)(1 + \mu p)$$

It can be seen from the ZIP mean and variance that when p equals zero the ZIP model reduces to the standard Poisson model. In contrast, as p approaches one the variance increases and the data exhibit greater overdisperion. The over-dispersion accounted for in the ZIP model is conceptually a result of the structural zeroes. Interpretation of the ZIP model depends upon what is being modeled.

Zero-Inflated Negative Binomial (ZINB)

Zero-inflated Negative Binomial models are sometimes preferred because they allow for additional flexibility in the variance. Using Eq. (2) we can express the ZINB model for participant i at dose j as

$$\Pr(Y_{ij} = y_{ij}) = \begin{cases} p_{ij} + (1 - p_{ij}) \dfrac{1}{(1 + a\mu_{ij})^{1/a}} & y_{ij} = 0 \\ (1 - p_{ij}) \dfrac{\Gamma(y_{ij} + 1/a)}{\Gamma(y_{ij} + 1)\Gamma(1/a)} \dfrac{(a\mu_{ij})^{y_{ij}}}{(1 + a\mu_{ij})^{y_{ij}+1/a}} & y_{ij} = 0 \end{cases} \quad (5)$$

where all terms have been defined previously and the mean is as for the ZIP model but the variance is given by $\sigma^2 = \mu(1 - p)[1 + \mu(p + \alpha)]$. Note that the variance depends on p and the dispersion parameter α. The ZINB model allows for added flexibility compared to the ZIP model. It allows for over-dispersion arising from excess zeroes and heterogeneity, whereas the ZIP model only accommodates overdispersion from excess zeroes. Interpretation of the ZINB model is as for the ZIP model.

Hurdle Models: Poisson and Negative Binomial

In contrast to zero-inflated models, hurdle models can be interpreted as twopart models. The first part is typically a binary response model and the second part is usually a truncated-at-zero count model (Cameron and Trivedi, 1998). Hence, the hurdle model is a modified count model in which separate processes generating the zeroes and positive counts are not constrained to be the same. This allows us to interpret the positive outcomes (> 0) that result from passing the zero hurdle (threshold). The hurdle portion of the two-part model estimates the probability that the threshold is crossed. Theoretically the threshold could be any value, but it’s usually taken as zero because this is most often meaningful in the context of the study objectives. Mullahy (1986) laid

out the basic form of hurdle count models. Assume that f_1 and f_2 are any probability density functions for nonnegative
integers. A hurdle model can be expressed as

$$P[y=0]=f_1(0)=p$$
$$P[y=i]=(1-p)\frac{f_2(i)}{1-f_2(0)}=(1-p)f_2'(i) \qquad i>0 \qquad (6)$$

Note that $f_1(\bullet)$ governs the hurdle part and $f_2(\bullet)$ the count process once the hurdle has been crossed. Furthermore, $f_1(0)$ is the probability of crossing the hurdle and $f_2'(\bullet)$ is the truncated normalization of $f_2(\bullet)$. Note that if $f_1(\bullet)=f_2(\bullet)$ the hurdle model collapses to the standard count model. Hurdle models can be specified in various ways by choosing different distributions for $f_1(\bullet)$and $f_2(\bullet)$ As for the zero-inflated models we use logistic regression to model p. Here we define two hurdle models by specifying f2_•_ as the Poisson and NB distributions. For example, substitution of Eq. (1) into Eq. (6) results in the Poisson hurdle model for participant i at dose j being defined as

$$\Pr(Y_{ij}=y_{ij})=\begin{cases} p_{ij} & y_{ij}=0 \\ (1-p_{ij})\dfrac{\mu_{ij}{}^{y_{ij}}}{(1-\exp(-\mu_{ij}))y_{ij}!}\exp(-\mu_{ij}), & y_{ij}=0 \end{cases} \qquad (7)$$

All terms are as defined previously and specification of the log-likelihood can be obtained using Eq. (7). The expected value for the Poisson hurdle (PH) model is given by

$$E(y)=\frac{(1-p)\mu}{1-f_2(0)}.$$

Substitution of Eq. (2) into Eq. (6) for $f_2(\bullet)$ results in the Negative Binomial hurdle (NBH) model. Computing the expected value for the NBH model is as for the PH model.

Score test

A score test first developed by Dean and Lawless (1989) to evaluate whether the amount of overdispersion in a Poisson model is sufficient to violate the basic assumptions of the model may be defined as:

$$Z_i=\frac{(y_i-\mu_i)^2-y_i}{\mu_i\sqrt{2}}$$

The test is post-hoc, i.e. it is performed subsequent to modeling the data.

Lagrange multiplier test

$$\mathrm{LM}=\frac{(\sum_{i=1}^{n}\mu_i^2-n\bar{y}_i)^2}{2\sum_{i=1}^{n}\mu_i^2}$$

The Lagrange multiplier test is evaluated using a chi2 test rather than on the t-test probability required for the score test.

Likelihood-ratio test

The likelihood-ratio (LR) test is a commonly used comparative fit test. It is generally used for nested models, but has also been used to test different models (e.g. whether data are better modeled using a negative binomial or a Poisson).
The traditional likelihood ratio test is defined as

$$\text{LR} = -\mathbf{2}\{\mathcal{L}_{\boldsymbol{reduced}} - \mathcal{L}_{\boldsymbol{full}}\}$$

where $\mathcal{L}_{\boldsymbol{full}}$ is the log-likelihood for a full or more complete model and $\mathcal{L}_{\boldsymbol{reduced}}$ is the log-likelihood for a reduced model.

Akaike and Bayesian Information Criterion

The Akaike Information Criterion (AIC) was developed by Hirotsugu Akaike in 1974. However, it did not begin to enjoy widespread use until the twenty-first century. It is now one of the most, if not the most, commonly used fit statistic displayed in statistical model output.
The second foremost contemporary comparative fit statistic for likelihood based statistical models is the Bayesian Information Criterion (BIC). Again, this statistic has undergone a variety of parameterizations. The original formulation was given by the University ofWashington's Adrian Raftery in 1986.

$$\text{AIC} = \frac{-2(\mathcal{L} - k)}{n}$$

$$\text{BIC} = \frac{-2(\mathcal{L} - k * \ln(k))}{n}$$

where $\mathcal{L}$ is the model log-likelihood, k is the number of predictors including the intercept, and n represents the number of model observations, A smaller AIC indicates a better fitted model.

Vuong test

The Vuong statistic proposed by Vuong (1989) for non-nested models is given by

$$V = \frac{\bar{m}\sqrt{n}}{S_n}$$

where $m_i = \ln\left[\frac{\widehat{P_1}(Y_i X_i)}{P_S\widehat{(Y_i|X_i)}}\right]$, P_s is usually taken to be the standard Poisson/NB and P_s is the zero-inflated/hurdle model. The statistic m_i has a mean $\bar{m}$ and standard deviation S_m. The statistic V asymptotically follows a standard normal distribution.

R code

```
library(xlsx)
library(MASS)
library(pscl)
library(lmtest)
library(vcdExtra)

p <- rstudioapi::getActiveDocumentContext()$path   # getting the path of current open file
setwd(dirname(p))                                  # set working directory to current open file location
## Importe Data ----
SData <- read.xlsx("ScorpData_Tggt.xlsx", 1)       # import excel file

# create new dataset with only variables of interest
# S: Daily recorded scorpion sting cases, Tm: Daily mean temperature,
#RH: Daily mean relative humidity, Wx: Daily max wind speed, tr=sin(trend*pi/365)
```

```
trnd <- sin(SData[, 2]*pi/365)
Scorp_Data <- data.frame( S = SData[1:1095,3] ,mT = SData[1:1095,4], RH = SData[1:1095,5],
                         Wx = SData[1:1095,6], tr= trnd[1:1095])

summary(Scorp_Data)                    # Summarize data
var(Scorp_Data$S)                      # variance of scorpion sting cases
var(Scorp_Data$S)/mean(Scorp_Data$S)   # variance to mean ratio
sd(Scorp_Data$S)                       # standard deviation
cor(Scorp_Data$S, Scorp_Data)          # correlation between S and (Tm, RH, Wx and tr)

# Observed scorpion stings count
scorp.fac <- factor(Scorp_Data$S, levels=0:24)
scorp.tab <- table(scorp.fac)
barplot(scorp.tab, xlab="Scorpion stings",
        ylab="Frequency",col="lightblue", ylim = c(0,280))
abline(v=mean(Scorp_Data$S), col="red", lwd=2) # mean

#### Fit Regression Models ----

# Dependent variable: S, Independent variables: Tm, RH, Wx and tr
# Fit a Poisson model with all the explanatory variables
P0 <- glm(formula = S ~ mT + RH + Wx + tr, family = poisson, data = Scorp_Data)
summary(P0)             # Summarize model P0
drop1(P0, test="Chisq")  # Compute variables can be dropped from the model
P1 <- glm(formula = S ~ mT + RH + tr, family = poisson, data = Scorp_Data) # fit a Poisson model without
predictor Wx
# Likelihood ratio test
lr <- -2*(logLik(P1)-logLik(P0)); lr
pchisq(lr, df= 1, lower.tail = F)              # Wx is not sinignificant predictor in the model

P <- P1                                  # choose the Poisson model without predictor Wx
pr <- sum(residuals(P, type="pearson")^2); pr        # Pearson Chi2
pr/P$df.residual

# check for overdispersion with Score or Lagrange multiplier test

# Score test for overdispersion   (Hilbe, Negative Binomial Regression, p175, 2011)
mu <- predict(P, type="response")
z <- ((Scorp_Data$S - mu)^2 - Scorp_Data$S)/ (mu * sqrt(2))
summary(zscore <- lm(z ~ 1))

#Lagrange multiplier test for overdispersion ((Hilbe, Negative Binomial Regression, p176, 2011))
mu <- predict(P, type="response")
mmu <- mean(mu)
nybar <- nrow(Scorp_Data)*mmu
musq <- mu^2
```

```
mu2 <- mean(musq)*nrow(Scorp_Data)
chival <- (mu2 - nybar)^2/(2*mu2)
chival
pchisq(chival,1,lower.tail = FALSE)

# Fit other models
# Negative binomial model
NB <- glm.nb(formula = S ~ mT + RH + tr, data = Scorp_Data)
# Zero-inflated Poisson model
ZIP <- zeroinfl(formula = S ~ mT + RH + tr, data = Scorp_Data, dist = "poisson")
# Zero-inflated Negative Binomial model
ZINB <- zeroinfl(formula = S ~ mT + RH + tr, data = Scorp_Data, dist = "negbin")
# Poisson Hurdle model
PH <- hurdle(formula = S ~ mT + RH + tr, data = Scorp_Data, dist = "poisson")
# Negative binomial Hurdle model
NBH <- hurdle(formula = S ~ mT + RH + tr, data = Scorp_Data, dist = "negbin")

# List of models

#### Goodness of fit measures ----
## Likelihood ratio test for nested models
# to determine if the over-dispersion parameter was significant
# P vs NB
lrtest(P, NB)
# ZIP vs ZINB
lrtest(ZIP, ZINB)
# PH vs NBH
lrtest(PH, NBH)

# test Overdispersion due excess zeros
# P vs ZIP
lr <- -2*(logLik(P) - logLik(ZIP)); lr
pchisq(lr, df= (length(coef(ZIP))-length(coef(P))), lower.tail = F)
# NB vs ZINB
lr <- -2*(logLik(NB) - logLik(ZINB)); lr
pchisq(lr, df= (length(coef(ZINB))-length(coef(NB))), lower.tail = F)

# Vuong test for non nested models
vuong(PH, P)
vuong(PH, ZIP)
vuong(NBH, NB)
vuong(NBH, ZINB)

# Log-Likelihood
logLik(P)
logLik(NB)
```

```
logLik(ZIP)
logLik(ZINB)
logLik(PH)
logLik(NBH)

# AIC and BIC
stats <- LRstats(P, NB, PH, NBH, ZIP, ZINB, sortby="AIC")
stats
write.xlsx(stats,"Results.xlsx", sheetName = "AIC_BIC", append = TRUE)

#### Compare observed and modeled counts ----
models.names <- c("Poisson", "NB", "ZIP", "ZINB", "PH", "NBH")
Obs.counts <- table(factor(Scorp_Data$S, levels=0:24))
Modeled.count <- list( )
Modeled.count[[1]] <- sapply(0:24 , function(x) sum(dpois(x, fitted(P))))
Modeled.count[[2]] <- sapply(0:24, function(x) sum(dnbinom(x, mu = fitted(NB), size = NB$theta)))
Modeled.count[[3]] <- colSums(predict(ZIP, type = "prob"))
Modeled.count[[4]] <- colSums(predict(ZINB, type = "prob"))
Modeled.count[[5]] <- colSums(predict(PH, type = "prob"))
Modeled.count[[6]] <- colSums(predict(NBH, type = "prob"))
names(Modeled.count) <- models.names
write.xlsx(Modeled.count,"Results.xlsx", sheetName = "Modeled.count", append = TRUE)

#### Compare observed and predicted counts ----
Scorp_NewData <- data.frame(  S = SData[1096:1339,3] ,mT = SData[1096:1339,4],
                              RH = SData[1096:1339,5],Wx = SData[1096:1339,6], tr=trnd[1096:1339])
newObs.counts <- table(factor(Scorp_NewData$S, levels=0:24))
Predicted.count <- list( )
Predicted.count[[1]] <- table(factor(Scorp_NewData$S, levels=0:24))
Predicted.count[[2]] <- sapply(0:24 , function(x) sum(dpois(x, predict(P, Scorp_NewData, type = "r" ))))
Predicted.count[[3]] <- sapply(0:24, function(x) sum(dnbinom(x, mu = predict(NB, Scorp_NewData, type =
"r" ), size = NB$theta)))
Predicted.count[[4]] <- colSums(predict(ZIP, Scorp_NewData, type = "prob"))
Predicted.count[[5]] <- colSums(predict(ZINB, Scorp_NewData, type = "prob"))
Predicted.count[[6]] <- colSums(predict(PH, Scorp_NewData, type = "prob"))
Predicted.count[[7]] <- colSums(predict(NBH, Scorp_NewData, type = "prob"))
names(Predicted.count) <- c("Obs.","P", "NB", "ZIP", "ZINB", "PH", "NBH")
write.xlsx(Predicted.count,"Results.xlsx", sheetName = "Predicted.count", append = TRUE)

#### Evaluation (RMSE & MAE)
test.data = Scorp_NewData[1:nrow(Scorp_NewData), names(Scorp_NewData)[names(Scorp_NewData)!= 'S']]

eval.rmse <- function(model, test.data, test.values) {
  output = predict(model, test.data, type="response")
  rmse = sqrt(sum((output - test.values)^2)/length(test.values))
}
```

```
eval.MAE <- function(model, test.data, test.values) {
  output = predict(model, test.data, type="response")
  rmse = sum(abs(output - test.values))/length(test.values)
}

models.names <- c("Poisson", "NB", "ZIP", "ZINB", "PH", "NBH")
Evaluate_RMSE <- list( )
Evaluate_RMSE[[1]] <- eval.rmse(P, test.data, Scorp_NewData$S)
Evaluate_RMSE[[2]] <- eval.rmse(NB, test.data, Scorp_NewData$S)
Evaluate_RMSE[[3]] <- eval.rmse(ZIP, test.data, Scorp_NewData$S)
Evaluate_RMSE[[4]] <- eval.rmse(ZINB, test.data, Scorp_NewData$S)
Evaluate_RMSE[[5]] <- eval.rmse(PH, test.data, Scorp_NewData$S)
Evaluate_RMSE[[6]] <- eval.rmse(NBH, test.data, Scorp_NewData$S)
names(Evaluate_RMSE) <- models.names
Evaluate_RMSE
write.xlsx(Evaluate_RMSE,"Results.xlsx", sheetName = "RMSE", append = TRUE)

models.names <- c("Poisson", "NB", "ZIP", "ZINB", "PH", "NBH")
Evaluate_MAE <- list( )
Evaluate_MAE[[1]] <- eval.MAE(P, test.data, Scorp_NewData$S)
Evaluate_MAE[[2]] <- eval.MAE(NB, test.data, Scorp_NewData$S)
Evaluate_MAE[[3]] <- eval.MAE(ZIP, test.data, Scorp_NewData$S)
Evaluate_MAE[[4]] <- eval.MAE(ZINB, test.data, Scorp_NewData$S)
Evaluate_MAE[[5]] <- eval.MAE(PH, test.data, Scorp_NewData$S)
Evaluate_MAE[[6]] <- eval.MAE(NBH, test.data, Scorp_NewData$S)
names(Evaluate_MAE) <- models.names
write.xlsx(Evaluate_MAE,"Results.xlsx", sheetName = "MAE", append = TRUE)

actual <- Scorp_NewData$S
predicted <- predict(NBH, Scorp_NewData, type="response")
ActPred <- data.frame(Actual= actual, Predicted= predicted )
write.xlsx(ActPred,"Results.xlsx", sheetName = "ActPred", append = TRUE)
```

R code Map

```
library("xlsx")
library("tmap")
library("tmaptools")
library("sf")
library("leaflet")
p <- rstudioapi::getActiveDocumentContext()$path   # getting the path of current open file
setwd(dirname(p))                                  # set working directory to current open file location

ExcelData <- read.xlsx(file="MapData.xlsx",1,header = TRUE) # Import the Excel file that contains the data
```

```
                                        #to be represented on the map.

ShapData <- read_shape(file="SHP/RIGH.shp", as.sf = TRUE )  # Import the shapefile

ExcelData$Municipalitie <-as.character(ExcelData$Municipalitie) # to compare shapefile objects and
ExcelData objects,
ShapData$COMMUNE <- as.character(ShapData$COMMUNE)  # must convert them into characters

ShapData <- ShapData[order(ShapData$COMMUNE),]     # to compare the name of municipality of shapefile
and that of ExcelData
ExcelData <- ExcelData[order(ExcelData$Municipalitie),]     # order them according to the municipality
(COMMUNE) column
                                        # in each file with the ordred () function.

identical(ShapData$COMMUNE,ExcelData$Municipalitie)      # check if the municipality (commune) name is
written in the same way in each file

MyMap <- (append_data(ShapData, ExcelData, key.shp = "COMMUNE",
             key.data = "Municipalitie" ))      # make a join between the two data structures ShapData and
                                        # Excel Data according to the key wilaya name

tmap_mode("plot")
breaks = c(3,4,5,6,7,8,9, 10)*100                # color breaks

#Making maps
map13 <- tm_shape(MyMap) +
  tm_fill("Incd.2013",breaks= breaks, palette = "Greens",title= "Incidence",id= "COMMUNE")+
  tm_borders() +
  tm_layout( title= "2013",title.size = 1,title.position = c(0.3,0.88), inner.margin= 0.1, frame = F,legend.title.size
= 0.9 , legend.text.size = 0.7)



map14 <- tm_shape(MyMap) +
  tm_polygons("Incd.2014",breaks= breaks, palette = "Greens",title= "Incidence",id= "COMMUNE")+
  tm_layout( title= "2014",title.size = 1, title.position = c(0.3,0.88), inner.margin= 0.1, frame = F, legend.show
= F)

map15 <- tm_shape(MyMap) +
  tm_polygons("Incd.2015",breaks= breaks, palette = "Greens",title= "Incidence",id= "COMMUNE")+
  tm_layout( title= "2015",title.size = 1, title.position = c(0.3,0.88), inner.margin= 0.1, frame = F, legend.show
= F) +  tm_compass(type = "8star", size = 3, position = c(0.65, 0.8)) +
  tm_scale_bar()

map <- tmap_arrange(map13, map14, map15)
map
tmap_save(map, "TGGT_map2 300 .tiff", dpi = 300)
```

Table 1. Demographic and epidemiological characteristics of patients stung by scorpions.

Year	2013					2014					2015					2016				
**Gender**	**F**	**M**	**M/F**	**TOTAL**	**%**	**F**	**M**	**M/F**	**TOTAL**	**%**	**F**	**M**	**M/F**	**TOTAL**	**%**	**F**	**M**	**M/F**	**TOTAL**	**%**
	**586**	**687**	**1.2**	**1273**		**538**	**717**	**1.3**	**1255**		**539**	**700**	**1.3**	**1239**		**393**	**552**	**1.4**	**945**	
**Age groups**																				
**< 1**	2	0	0	2	0.2	1	1	1	2	0.2	1	0	0	1	0.1	3	0	0	3	0.3
**1 - 3**	17	8	0.5	25	2.0	18	17	0.9	35	2.8	9	13	1.4	22	1.8	12	20	1.7	32	3.4
**4 - 5**	15	12	0.8	27	2.1	12	16	1.3	28	2.2	14	13	0.9	27	2.2	9	14	1.6	23	2.4
**6 - 9**	23	26	1.1	49	3.8	17	25	1.5	42	3.3	23	26	1.1	49	4.0	24	27	1.1	51	5.4
**10 - 19**	115	148	1.3	263	20.7	100	133	1.3	233	18.6	91	135	1.5	226	18.2	64	95	1.5	159	16.8
**20 - 29**	121	145	1.2	266	20.9	110	209	1.9	319	25.4	123	171	1.4	294	23.7	83	152	1.8	235	24.9
**30 - 39**	99	117	1.2	216	17.0	102	122	1.2	224	17.8	91	110	1.2	201	16.2	63	82	1.3	145	15.3
**40 - 49**	49	79	1.6	128	10.1	63	55	0.9	118	9.4	70	79	1.1	149	12.0	45	59	1.3	104	11.0
**50 - 59**	62	52	0.8	114	9.0	41	49	1.2	90	7.2	54	56	1.0	110	8.9	41	40	1.0	81	8.6
**60 - 69**	38	40	1.1	78	6.1	31	33	1.1	64	5.1	27	47	1.7	74	6.0	24	38	1.6	62	6.6
**70 - 79**	12	29	2.4	41	3.2	10	28	2.8	38	3.0	22	28	1.3	50	4.0	13	13	1.0	26	2.8
**80 - 89**	8	10	1.3	18	1.4	7	8	1.1	15	1.2	5	11	2.2	16	1.3	1	5	5.0	6	0.6
**≥ 90**	2	2	1.0	4	0.3	2	2	1.0	4	0.3	0	2	--	2	0.2	2	0	0.0	2	0.2
**Unknown**	23	19	0.8	42	3.3	24	19	0.8	43	3.4	9	9	1.0	18	1.5	9	7	0.8	16	1.7
**Anatomical sting site**																				
**Lower Limbs**	286	371	1.3	657	51.6	258	395	1.5	653	52.0	265	412	1.6	677	54.6	189	289	1.5	478	50.6
**Upper Limbs**	240	252	1.1	492	38.6	218	242	1.1	460	36.7	235	249	1.1	484	39.1	162	196	1.2	358	37.9
**Head/Neck**	39	32	0.8	71	5.6	38	41	1.1	79	6.3	24	13	0.5	37	3.0	19	28	1.5	47	5.0
**Trunk**	12	14	1.2	26	2.0	19	35	1.8	54	4.3	13	22	1.7	35	2.8	21	36	1.7	57	6.0
**Unknown**	9	18	2.0	27	2.1	5	4	0.8	9	0.7	2	4	2.0	6	0.5	2	3	1.5	5	0.5
**Grade on first clinical examination**																				
**Mild**	529	607	1.1	1136	89.2	479	628	1.3	1107	88.2	527	686	1.3	1213	97.9	375	540	1.4	915	96.8
**Moderate**	19	24	1.3	43	3.4	38	69	1.8	107	8.5	8	6	0.8	14	1.1	12	11	0.9	23	2.4
**Severe**	1	0	0.0	1	0.1	0	1	--	1	0.1	0	1	--	1	0.1	4	0	0.0	4	0.4
**Unknown**	37	56	1.5	93	7.3	21	19	0.9	40	3.2	4	7	1.8	11	0.9	2	1	0.5	3	0.3
**Sing time**																				
**0 - 5**	124	128	1.0	252	19.8	93	142	1.5	235	18.7	107	116	1.1	223	18.0	90	145	1.6	235	24.9
**6 - 11**	192	238	1.2	430	33.8	186	232	1.2	418	33.3	192	251	1.3	443	35.8	113	133	1.2	246	26.0
**12 - 17**	152	169	1.1	321	25.2	130	138	1.1	268	21.4	109	142	1.3	251	20.3	62	99	1.6	161	17.0
**18 - 23**	99	135	1.4	234	18.4	111	187	1.7	298	23.7	120	177	1.5	297	24.0	123	169	1.4	292	30.9
**Unknown**	19	17	0.9	36	2.8	18	18	1.0	36	2.9	11	14	1.3	25	2.0	5	6	1.2	11	1.2
**Location**																				
**Inside dwellings**	451	391	0.9	842	66.1	472	463	1.0	935	74.5	418	443	1.1	861	69.5	326	278	0.9	604	63.9
**Outside dwellings**	111	273	2.5	384	30.2	42	226	5.4	268	21.4	114	252	2.2	366	29.5	58	269	4.6	327	34.6
**Unknown**	24	23	1.0	47	3.7	24	28	1.2	52	4.1	7	5	0.7	12	1.0	9	5	0.6	14	1.5
